# Network Pharmacology and Molecular Docking Analysis of Shufeiya Recipe in the Treatment of Pulmonary Hypertension

**DOI:** 10.1155/2022/7864976

**Published:** 2022-12-28

**Authors:** Zeyu Zhang, Xianliang Wang, Shuai Wang, Zhuangzhuang Jia, Jingyuan Mao

**Affiliations:** First Teaching Hospital of Tianjin University of Traditional Chinese Medicine, National Clinical Research Center for Chinese Medicine Acupuncture and Moxibustion, Tianjin 300381, China

## Abstract

**Objective:**

This study is aimed at exploring the molecular mechanism of Shufeiya recipe in the treatment of pulmonary hypertension (PH) using network pharmacology and molecular docking analysis.

**Methods:**

Active components and their target proteins in the recipe were screened using the TCMSP database. PH-related core proteins were screened using GeneCards, STRING database, and Cytoscape-v3.8.2. Common proteins were obtained by intersection of the target proteins of these recipe active components and pH-related core proteins. Rx64 4.0.2 software was used to perform GO functional enrichment analysis and KEGG pathway enrichment analysis on the common proteins to obtain pathway-enriched proteins, and then core enriched proteins were further screened. We analyzed the relationship between the active components and pathway-enriched proteins using Cytoscape-v3.8.2. AutoDock Vina was used to dock their core proteins into the components.

**Results:**

Shufeiya recipe contained 67 active components. 61 common proteins of the target proteins of the active components and PH-related core proteins were obtained. The treatment involved both functional and pathway regulations. The core pathway-enriched proteins were prostaglandin G/H synthase 2 (PTGS2), epidermal growth factor receptor (EGFR), and RAC-alpha serine/threonine-protein kinase (AKT1), and their binding energies to the corresponding components were all less than -5 kJ•mol-1.

**Conclusion:**

It was found that the main mechanism might be the active components acting on the core pathway-enriched proteins to regulate related signaling pathways, thereby playing roles in anticoagulation, vasodilation, anti-PASMC proliferation, promotion of PAECs apoptosis, inhibition of oxidative stress, and anti-inflammatory effects.

## 1. Introduction

Pulmonary hypertension (PH) is a chronic progressive pulmonary vascular system disease caused by pulmonary arteriole obstruction, and it is characterized by pulmonary artery vasoconstriction and remodeling, increased pulmonary circulatory resistance, and increased pulmonary artery pressure (mean pulmonary artery pressure at rest ≥ 25 mmHg). Its clinical manifestations include chest tightness, shortness of breath, fatigue, and dyspnea, which can eventually lead to right-sided heart failure and death [1]. Epidemiological surveys show that about 1% of people suffer from PH around the world, about 10% of people over 65 years old suffer from PH, the 1-year mortality rate of this disease is about 15%, and the 3-year mortality rate is about 30% [2, 3]. Therefore, it is important to seek effective methods for the prevention and treatment of PH.

The clinical practice of PH treatment in our country not only fully applies modern research results but also recognizes that traditional Chinese medicine (TCM) has the synergistic effects of improving clinical symptoms and quality of life, increasing exercise tolerance, and even improving prognosis. The combination of Chinese and western medicine has become a generally accepted treatment strategy for PH in our country. Based on his clinical practice and researches of many years, Professor Mao Jingyuan, as a “Qihuang Scholar,” and national leading talent of TCM, concludes that Shufeiya recipe can be used for the treatment of PH; this recipe is composed of platycodi radix (Jiegeng), *Salvia miltiorrhiza* (Danshen), safflower (Honghua), and *Cornus officinalis* (Shanzhuyu). Previous animal studies suggested that Shufeiya recipe could downregulate serum reactive oxygen species levels in rats with pulmonary hypertension induced by monocrotaline; upregulate serum NO, sGC, cGMP, PKG, PTGS1, PTGS2, and Mn-SOD activity; inhibit oxidative damage; promote the expression of SIRT3, FOXO3a, p-PI3K, p-AKT, and p-eNOS protein in the pulmonary artery; downregulate the protein expression of Ras, p-MEK1/2, p-ERK1/2, and c-fos in the pulmonary artery; and reduce the thickening of the pulmonary vascular wall and pulmonary artery pathological injury such as cavity stenosis, thereby reducing pulmonary artery pressure in the model group [4, 5]. However, the specific mechanism of action of the Shufeiya recipe has not been fully elucidated, and needs to be explored using more analytical methods and from various perspectives. Therefore, this study is aimed at exploring the molecular mechanism of Shufeiya recipe in the treatment of pulmonary hypertension (PH) using network pharmacology and molecular docking analysis.

## 2. Materials and Methods

### 2.1. Active Components Screening and their Target Proteins Obtained

Based on the Traditional Chinese Medicine Systems Pharmacology Database and Analysis Platform (TCMSP) (http://tcmspw.com/tcmsp.php) [6] and the screening criteria of oral bioavailability (OB) ≥ 30%, drug − likeness (DL) ≥ 0.18, octanol − water partition coefficient (logP) ≤ 5, hydrogen bond donors (Hdon) ≤ 5, hydrogen bond acceptors (Hacc) ≤ 10, and relative molecular weight (MW) <500, the active components and their target proteins of platycodi radix, *Salvia miltiorrhiza*, safflower, and *Cornus officinalis* were screened, and the target proteins were imported into the UniProt database (https://www.uniprot.org/) [7] to standardize the proteins.

### 2.2. The “Drug-Disease” Targets Obtained

Based on the GeneCards database (http://www.GeneCards.org/) [8], PH-related proteins were obtained, with PH as the search term and a relevance score of >6 as the screening criteria. The STRING database (http://string-db.org) [9] was used to predict protein-protein interactions with the criterion of medium confidence = 0.4. The Cytoscape-v3.8.2 software [10] was used to screen PH-related core proteins, with the condition that degree, betweenness, closeness, network, and local marginal connectivity (LAC) were all greater than the median value. Finally, the intersection of “PH-related core proteins” and “target proteins of the active components” was used to obtain the common targets, named “drug-disease” targets.

### 2.3. Gene Ontology (GO) Functional Enrichment Analysis and Kyoto Encyclopedia of Genes and Genomes (KEGG) Pathway Enrichment Analysis

The “drug-disease” targets were subjected to GO functional enrichment analysis, and KEGG pathway enrichment analysis using Rx64 4.0.2 software [11], with *P* < 0.05 as the screening criteria. GO functional enrichment analysis mainly included three aspects: biological processes (BP), molecular functions (MF), and cellular components (CC). The top ten functions obtained in each aspect by GO enrichment analysis were visualized using a bar chart. The top 30 signaling pathways obtained by KEGG pathway enrichment analysis were visualized using a bubble chart, where the ordinate represents the pathway name, the abscissa represents the gene ratio, the bubble size represents the number of enriched proteins, the color represents the degree of enrichment, and the darker the red, the stronger the enrichment. PH-related pathways were screened by referring to the literature, and enriched proteins were obtained from KEGG pathway enrichment analysis. The map of the pathways most significantly related to the treatment of PH by Shufeiya recipe was obtained from the KEGG database (https://www.kegg.jp/) [12]. The red nodes in this map represent the pathway-enriched proteins.

### 2.4. Screening of Core Enriched Proteins, and Construction of the Pathway-Target Topology Network

The enriched proteins of PH-related pathways were imported into the STRING database, and Cytoscape-v3.8.2 software was used to screen the core enriched proteins of PH-related pathways with the condition that the degree, betweenness, closeness, network, and LAC were all greater than the median value. Then, PH-related pathways and these enriched proteins were imported into Cytoscape-v3.8.2 software for topology analysis and visualization, and a pathway-target topology network was constructed.

### 2.5. Construction of the Component-Target Topology Network

The active components of Shufeiya recipe and pathway-enriched proteins were imported into Cytoscape-v3.8.2 software for topology analysis and visualization, and the component-target topology network was constructed, where nodes represented components and targets, and edges represented interactions between nodes.

### 2.6. Molecular Docking Verification and Screening

Molecular docking was performed with the core-enriched proteins as the receptor, and the corresponding drug components as the ligand. The 2D structure of the drug component was downloaded from the PubChem database (https://pubchem.ncbi.nlm.nih.gov/) [13] and imported into the Chem3D software to be converted into a 3D structure. Then, we selected the Minimize Energy of the MM2 item in the calculations to optimize the component structure, and saved it as lig.mol2. The protein entry was obtained from the UniProt database (https://www.uniprot.org/), and entered into the PDB database (http://www.rcsb.org/) [14] to obtain the protein crystal structure; then the protein crystal structure was imported into PyMOL 2.5 software [15], “remove solvent and remove organic” item was used to remove its H2O and small molecule ligands, and the file was saved as rep.pdb. We imported rep.pdb into AutoDockTools 1.5.6 software [16], added Hydrogens, and saved it in the PDB format. We selected Macromolecule of the Grid item to output the protein in rep.pdbqt format; we clicked ligand to import lig.mol2 and output it as lig.pdbqt format. We selected the secondary structure and undisplay lines of the display item to convert the protein and the component into the secondary structure, and then selected the Grid BOX to determine the binding site of protein and component using the number of points in x − dimension = 40, number of points in y − dimension = 40, number of points in z − dimension = 40, spacing = 1 as conditions, and the output file was saved as grid.gpf. We saved rep.pdbqt, lig.pdbqt, and grid.gpf in the same folder, and then vina.exe, vina_split.exe, config.txt (receptor = rep.pdbqt, ligand = lig.pdbqt, center_x =, center_y =, center_z =, size_x = 40, size_y = 40, size_z = 40, energy_range = 5, and num_modes = 20) were saved in this folder, and center_x, center_y, and center_z of config.txt were filled with the gridcenter data of the grid.gpf file. The path of the folder was entered into Perl, and then the command “vina.exe --config config.txt --log log.txt --out output.pdbqt” was entered to calculate affinity. Twenty conformations were generated for docking results. The conformation with the best affinity was selected as the final docking conformation and was visualized using PyMOL 2.5.

## 3. Results

### 3.1. Active Components of Shufeiya Recipe and their Target Proteins

A total of 67 active components of Shufeiya recipe were obtained from the TCMSP database, including 3 active components from platycodi radix, 52 active components from *Salvia miltiorrhiza*, 10 active components from safflower, and 4 active components from *Cornus officinalis* (Supplementary Table 1). After protein prediction and standardization, 214 target proteins were identified.

### 3.2. PH-Related Core Proteins Screening and the “Drug-Disease” Targets Obtained

**Figure 1:**
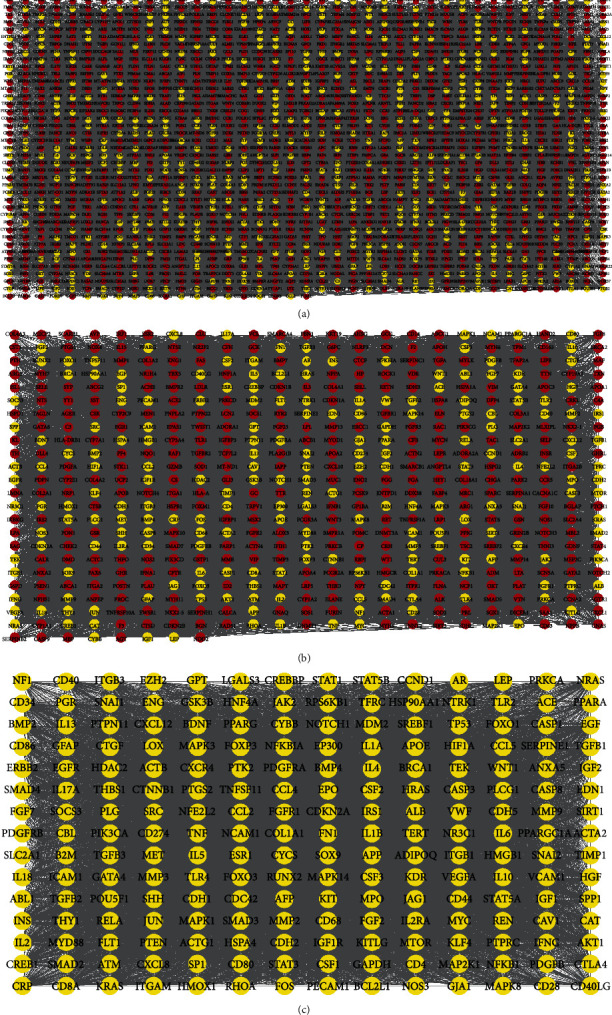
(a–c) The PH-related core protein screening process.

**Figure 2:**
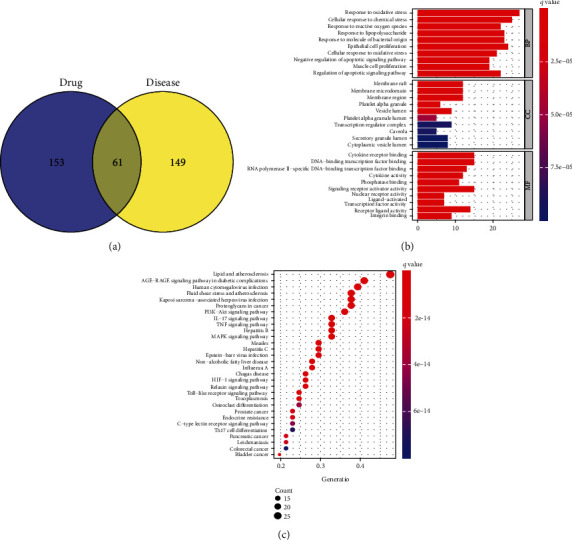
(a) Venn diagram of the intersection of PH-related core proteins and drug target proteins. (b) The bar plot of Go functional enrichment analysis. (c) The bubble plot of KEGG pathway enrichment analysis.

The PH-related core protein screening process was visualized using GeneCards, STRING database and Cytoscape-v3.8.2 software (Figures 1(a)–1(c)). The red nodes in figure 1(a)–1(c) represents the proteins whose degree, betweenness, closeness, network, and LAC were all less than the median value, and the yellow nodes represent the proteins whose degree, betweenness, closeness, network, and LAC were all greater than the median value.  Figure 1(a) shows the first screening result, Figure 1(b) shows the second screening result, and Figure 1(c) shows the PH-related core proteins. A total of 210 PH-related core proteins were obtained. After taking the intersection of 210 PH-related core proteins and 214 target proteins of drug active components, 61 common proteins (“drug-disease” targets) were obtained (Figure 2(a), Table 1).

### 3.3. GO and KEGG Enrichment Analyses

**Figure 3:**
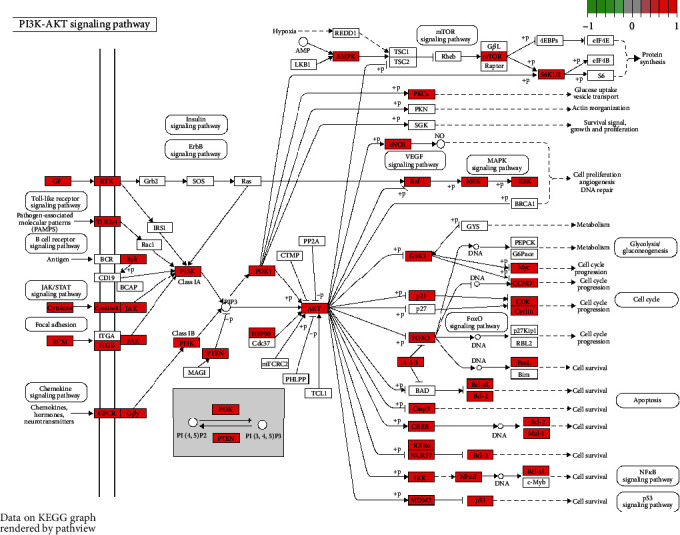
The map of PI3K-Akt pathway regulated by Shufeiya recipe.

A total of 2469 functional terms were obtained by GO functional enrichment analysis, including 2337 terms for BP, 299 terms for CC, and 100 terms for MF. The top ten functional terms for BP, MF and CC are shown in Figure 2(b). BP mainly included response to oxidative stress, cellular response to chemical stress, and epithelial cell proliferation; CC mainly included membrane raft membrane rafts, membrane microdomain, and membrane region; MF mainly included cytokine receptor binding, DNA-binding transcription factor binding, and signaling receptor activator activity. A total of 161 signaling pathways were obtained by the KEGG pathway enrichment analysis. The top 30 signaling pathways are shown in Figure 2(c). Previous studies have reported that the PI3K-AKT signaling pathway [17], IL-17 signaling pathway [18], TNF pathway [19], MAPK signaling pathway [20], HIF-1 signaling pathway [21], and Toll-like receptor signaling pathway [22] were related to the occurrence of PH, among which the PI3K-AKT pathway was most closely related to the treatment of PH with Shufeiya recipe (Figure 3).

### 3.4. Core-Enriched Proteins Screening and Pathway-Target Topology Network Analysis

**Figure 4:**
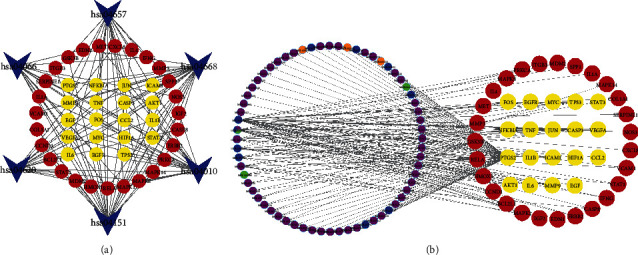
(a) pathway-target topology network analysis. (b) Component-target topology network analysis.

KEGG enrichment analysis showed that 46 proteins were enriched in the signaling pathways related to the treatment of pH by the Shufeiya recipe (Table 2). Based on the STRING database and Cytoscape-v3.8.2 software, these signaling pathways and enriched proteins were utilized to construct the pathway-target topology network for screening 19 core-enriched proteins (Figure 4(a)). In Figure 4(a), the blue arrows represent the signaling pathways related to the treatment of PH by Shufeiya recipe, the red nodes represent general enriched proteins, and the yellow nodes represent core enriched proteins, suggesting that prostaglandin G/H synthase 2 (PTGS2), epidermal growth factor receptor (EGFR), RAC-alpha serine/threonine-protein kinase (AKT1), vascular endothelial growth factor A (VEGFA), mitogen-activated protein kinase 1 (MAPK1), interleukin-6 (IL6), and receptor tyrosine-protein kinase erbB-2 (ERBB2) might play key roles in regulating these signaling pathways. Degree value refers to the number of pathway-enriched proteins. The PI3K-Akt signaling pathway was 22, the IL-17 signaling pathway was 20, the TNF signaling pathway was 20, the MAPK signaling pathway was 20, the HIF-1 signaling pathway was 16, and the Toll-like receptor signaling pathway was 15, indicating that the PI3K-Akt signaling pathway was the most regulated protein by Shufeiya recipe.

### 3.5. Component-Target Topology Network Analysis

The component-target topology network analysis showed that the network contained 110 nodes, including 64 nodes for drug components, 46 nodes for target proteins, and 167 edges. According to the data obtained by topological analysis, the components of Shufeiya recipe with degree ≥ 2 are listed in Table 3, which indicates that the main components of this recipe for the treatment of PH were quercetin, luteolin, baicalein, kaempferol, tanshinone IIA, and cryptotanshinone. The component-target topology network is shown in Figure 4(b). The left circle in Figure 4(b) represents the active components of this recipe, where green nodes represented components from platycodi radix, purple nodes represent components from *Salvia miltiorrhiza*, blue nodes represent components from safflower, orange nodes represent components from *Cornus officinalis*, and nodes of multiple colors represent the common components of multiple drugs. The right circle in figure 4(b) represents proteins enriched in the signaling pathways related to the treatment of PH by Shufeiya recipe, where the red nodes represent the general enriched proteins, and the yellow nodes represent the core enriched proteins. The degree value refers to the number of active components of the regulatory proteins. Among the core enriched proteins, PTGS2 was 63, CASP3 was 6, TP53 was 5, AKT1 was 4, TNF was 4, JUN was 4, MMP9 was 4, FOS was 3, and ICAM1 was 3, VEGFA was 3, NFKBIA was 3, EGFR was 2, IL6 was 2, HIF1A was 2, MYC was 2, CCL2 was 1, IL1B was 1, STAT3 was 1, and EGF was 1, which indicated the core protein PTGS2 was most strongly regulated by the components of this recipe.

### 3.6. Molecular Docking of these Core Enriched Proteins and the Drug Active Components

**Figure 5:**
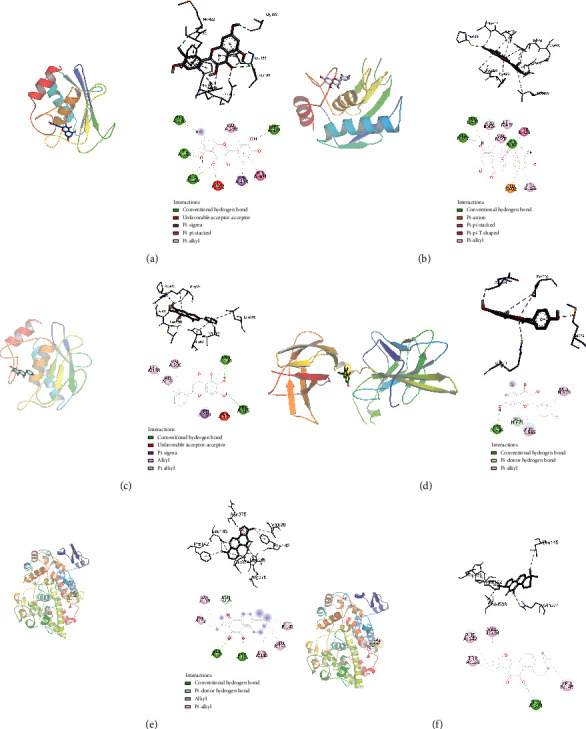
The molecular docking models. (a) The molecular docking model of quercetin and MMP9, (b) the molecular docking model of luteolin and MMP9. (c) The molecular docking model of baicalein and MMP9.. (d) The molecular docking model of kaoneferol and JUN. (e) The molecular docking model of tanshinone IIA and PTGS2. (f) The molecular docking model of cryptotanshinone and PTGS2.

When the binding energy between the receptor and ligand is lower than -5 kJ·mol^−1^, it indicates that the active component can bind well to the target protein [23]. The docking accuracy of the core enriched protein and its corresponding components was verified through molecular docking. The results showed that the binding energies of the core-enriched proteins and their corresponding components were all less than -5 kJ•mol^−1^ (Table 4). As shown in figure 5, a molecular docking model was constructed for the component and its target protein with the strongest affinity.  Figure 5(a) shows the molecular docking model of quercetin and MMP9, Figure 5(b) shows the molecular docking model of luteolin and MMP9, Figure 5(c) shows the molecular docking model of baicalein and MMP9, Figure 5(d) shows the molecular docking model of kaoneferol and JUN, Figure 5(e) shows the molecular docking model of tanshinone IIA and PTGS2, and Figure 5(f) shows the molecular docking model of cryptotanshinone and PTGS2. This indicated that there was good binding ability between the drug components and their targets.

## 4. Discussion

The occurrence of PH is closely related to abnormalities in the pulmonary vascular function and/or structure. Long-term hypoxia or other factors damage the endothelial cell structure, resulting in an imbalance of vasoactive substances, such as NO, ion channel dysfunction, and abnormal endothelial cell barrier function, which eventually leads to abnormal contraction of pulmonary blood vessels, progressive stenosis, and occlusion of the lumen. Its pathological characteristics include pulmonary artery intima damage and proliferation, media hypertrophy, adventitial matrix deposition fibrosis and inflammatory infiltration, distal vasodilation, and in situ thrombus [1]. Traditional Chinese medicine (TCM) is widely used for the treatment of PH because of its unique efficacy and safety. Modern science has thoroughly studied the mechanism of action of the components of traditional Chinese medicine to promote their application, so that Chinese and Western medicines complement each other. After years of clinical practice, traditional Chinese medicine believes that “stasis and bloking of lung and heart” is the pathogenesis of PH, Shufeiya recipe has functions of dispersing lung and removing stasis, and nourishing yin and dredging pulse, and the clinical effects of this recipe are quite good. Network pharmacology can clarify the mechanism of action of drugs at multiple levels, and molecular docking can be used to calculate the affinity between receptors and ligands. Therefore, the present study used network pharmacology and molecular docking methods to explore the relationship between Shufeiya recipe and PH, and preliminarily explored the specific mechanisms of functions, signaling pathways, components, and targets involved in the treatment of PH using Shufeiya recipe.

GO functional enrichment analysis showed that Shufeiya recipe for treating PH involved the regulation of functions such as oxidative stress, chemical stress, epithelial cell proliferation, and signaling receptor agonist activity. Oxidative stress is one of the key causes of PH, and is a process of oxidative damage caused by the imbalance between the generation and metabolism of oxygen free radicals in tissue cells, resulting in the accumulation of reactive oxygen species (ROS) and an imbalance in the ratio of antioxidant enzymes to lipid peroxides [24, 25]. ROS can activate or inhibit a series of signaling pathways, resulting in pathological changes, such as pulmonary artery smooth muscle cells (PASMCs) proliferation and migration, pulmonary artery endothelial cells (PAECs) disorders, situ thrombosis, inflammation, pulmonary vascular remodeling, vasoconstriction response, and extracellular matrix deposition [24, 25]. Our previous study confirmed that Shufeiya recipe could downregulate the serum level of ROS, upregulate PTGS1 and PTGS2 concentrations, and Mn-SOD activity, and inhibit oxidative damage [5]. KEGG pathway enrichment analysis showed that Shufeiya recipe could inhibit the development of PH by regulating PI3K-AKT, IL-17, TNF, MAPK, HIF-1, and Toll-like receptor signaling pathways. Activation of the PI3K-AKT signaling pathway can inhibit hypoxia-induced PASMCs cycle progression, proliferation, migration, and autophagy; regulate PASMCs cytoskeletal rearrangement, and phenotypic transition; protect PAECs from hypoxia-induced apoptosis; inhibit hypoxia-induced pulmonary vascular remodeling; and promotes angiogenesis and survival, thereby attenuating pulmonary vascular remodeling, and the development of PH [17]. Our previous study confirmed that Shufeiya recipe can promote the protein expression of p-PI3K and p-AKT [5]. Activation of the IL-17 signaling pathway can induce vascular oxidase, endothelial nitric oxide synthase, vascular smooth muscle cells, and adventitial fibroblast dysfunction [18]. Overexpression of TNF-*α* can promote the inflammatory response in rats with PH and reduce the expression of vascular endothelial growth factor and flk-1, causing pulmonary vascular remodeling [19]. The MAPK signaling pathway mainly includes ERK1/2, p38MAPK, and JNK, and hypoxia-induced activation of Ca^2+^-dependent PKC/MAPK signaling pathway in PH promotes PASMCs proliferation [26]. PH caused by left heart disease can promote the expression of matrix metalloproteinase 9 and transforming growth factor *β*-1 proprotein by activating the MAPK signaling pathway, which is involved in pulmonary artery vascular remodeling [27]. p38 MAPK*α*-related protein inhibition can effectively reduce inflammation, increase NO production, reduce superoxide load, inhibit pulmonary artery fibroblast proliferation, restore PAECs function, and reverse pulmonary vascular remodeling [28]. ERK activation can promote the proliferation of pulmonary artery fibroblasts, while Shufeiya recipe can downregulate the expression levels of Ras, p-MEK1/2, p-ERK1/2, and c-fos proteins in the ERK signaling pathway, thereby inhibitting PAECs fibrosis [5]. Activation of the HIF-1 signaling pathway in PAECs can promote the proliferation of pulmonary artery medial cells, and the expression of factors such as endothelin 1 [21, 29]. The Toll-like receptor signaling pathway is closely related to the proliferation, apoptosis, migration, and interstitial transition of PAECs [22, 30]. For example, inhibition of Toll-like receptor 3 can promote caspase-dependent PAECs apoptosis, and inhibition of Toll-like receptor 4 can limit PASMCs proliferation and migration [22, 30]. Screening of core-enriched proteins, pathway-target topology analysis, component-target topology analysis, and molecular docking suggested that quercetin, luteolin, baicalein, kaempferol, tanshinone IIA, and cryptotanshinone, as the main components of Shufeiya recipe, might effectively intervene in core proteins, including PTGS2, EGFR, AKT1, VEGFA, and MAPK1, to regulate their signaling pathways. Modern pharmacology suggests that quercetin can improve pulmonary arterial pressure, right ventricular hypertrophy, and vascular remodeling through anticoagulation, vasodilator, antiproliferation, and induction of PASMCs apoptosis [31]; luteolin can downregulate platelet-derived growth factor-BB-induced AKT phosphorylation, thereby inhibiting the proliferation and migration of PASMCs [17]; baicalein can block the production of 12(S)-hydroxyeicosatetraenoic acid to resist ERK1/2 phosphorylation, and inhibit hypoxia-induced proliferation of PASMCs [32]; kaempferol and its derivatives have a wide range of pharmacological activities, including anti-inflammatory, antibacterial, antioxidant, and relaxation of pulmonary arteries in an endothelium-independent manner [33]; tanshinone IIA can activate the PI3K/Akt/mTOR signaling pathway, reduce oxidative stress, inhibit inflammatory response, and downregulate the expression of high mobility group box 1 protein, thereby inhibiting the proliferation of PAECs and inflammatory response; in addition, its water-soluble derivative of tanshinone IIA sodium sulfonate can relax blood vessels on this basis [34–36]; and cryptotanshinone has anti-inflammatory effects, and inhibits epithelial-mesenchymal transition [37, 38]. Therefore, the Shufeiya recipe for the treatment of PH might have multifunction, multichannel, multicomponent, and multitarget advantages.

However, there are some limitations in this study. Our results were derived only from the analysis and prediction of the public database and *silicon* computers, and predicting herb targets only used one platform (TCMSP). Therefore, we will explore more detailed information about the recipe by searching a few more platform, such as BATMAN-TCM, Herb, and TCMID, and in vivo and in vitro experiments need to be performed for further validation in the future.

In summary, the molecular mechanism of Shufeiya recipe in the treatment of PH was analyzed using network pharmacology. We screened the active components and their targets of Shufeiya recipe in the treatment of PH, constructed component-target networks, analyzed their biological process annotations, and signaling pathways, and systematically studied the potential mechanism of Shufeiya recipe in treating PH. It was found that the main mechanism of this recipe in the treatment of PH might be that the active components of this recipe, including quercetin, luteolin, baicalein, kaempferol, tanshinone IIA, and cryptotanshinone, acted on the core proteins, including PTGS2, EGFR, AKT1, VEGFA, and MAPK1, to regulate signaling pathways such as PI3K-AKT, IL-17, TNF, MAPK, HIF-1, and Toll-like receptors, thereby playing the roles of anticoagulation, vasodilator, anti-PASMC proliferation, promotion of PAEC apoptosis, inhibition of oxidative stress, and anti-inflammatory effects. Our present study identified the core targets of Shufeiya recipe in the treatment of PH at the molecular level, clarified its potential material basis and mechanism, and provided a general reference for the clinical application and experimental research of traditional Chinese medicine in the treatment of PH. However, these results need to be confirmed by more experiments and clinical practices in the future.

## Figures and Tables

**Table 1:** The intersection of 210 PH-related core proteins and 214 target proteins of drug active components.

“Drug-disease” targets					
PTGS2	MMP2	APP	NR3C1	CYCS	IL1B
ESR1	MMP9	ERBB2	PGR	MAPK8	CCL2
AR	MAPK1	HMOX1	STAT3	STAT1	CXCL8
PPARG	TNF	ICAM1	EDN1	VCAM1	NOS3
RELA	JUN	IFNG	FOS	MMP3	SERPINE1
EGFR	IL6	IL4	MYC	EGF	COL1A1
AKT1	CASP3	CD40LG	ITGB3	CASP8	
VEGFA	TP53	MET	HIF1A	PRKCA	
CCND1	NFKBIA	MAPK14	MPO	CAV1	
BCL2L1	MDM2	GSK3B	IGF2	GJA1	
RUNX2	SPP1	PPARA	NFE2L2	IL1A	

**Table 2:** The enriched proteins of signaling pathways related to Shufeiya recipe treating PH.

ID	Description	Protein				
hsa04151	PI3K-Akt signaling pathway	RELA	EGFR	AKT1	VEGFA	CCND1
		BCL2L1	MAPK1	IL6	TP53	MDM2
		ERBB2	IL4	MET	GSK3B	MYC
		ITGB3	IGF2	EGF	PRKCA	NOS3
		COL1A1	SPP1			

hsa04657	IL-17 signaling pathway	PTGS2	RELA	MMP9	MAPK1	TNF
		JUN	IL6	CASP3	NFKBIA	IFNG
		IL4	MAPK14	GSK3B	FOS	MAPK8
		MMP3	CASP8	IL1B	CCL2	CXCL8

hsa04668	TNF signaling pathway	PTGS2	RELA	AKT1	MMP9	MAPK1
		TNF	JUN	IL6	CASP3	NFKBIA
		ICAM1	MAPK14	EDN1	FOS	MAPK8
		VCAM1	MMP3	CASP8	IL1B	CCL2

hsa04010	MAPK signaling pathway	RELA	EGFR	AKT1	VEGFA	MAPK1
		TNF	JUN	CASP3	TP53	ERBB2
		MET	MAPK14	FOS	MYC	IGF2
		MAPK8	EGF	PRKCA	IL1B	IL1A

hsa04066	HIF-1 signaling pathway	RELA	EGFR	AKT1	VEGFA	MAPK1
		IL6	ERBB2	HMOX1	IFNG	STAT3
		EDN1	HIF1A	EGF	PRKCA	NOS3
		SERPINE1				

hsa04620	Toll-like receptor signaling pathway	RELA	AKT1	MAPK1	TNF	JUN
		IL6	NFKBIA	MAPK14	FOS	MAPK8
		STAT1	CASP8	IL1B	CXCL8	SPP1

**Table 3:** Topological analysis of components in the Shufeiya recipe.

Component ID	Component name	Degree	Drug
MOL000098	Quercetin	37	Safflower
MOL000006	Luteolin	22	*Salvia miltiorrhiza*/safflower/Platycodi radix
MOL007154	Tanshinone II a	11	*Salvia miltiorrhiza*
MOL000422	Kaempferol	11	Safflower
MOL002714	Baicalein	10	Safflower
MOL007088	Cryptotanshinone	7	*Salvia miltiorrhiza*
MOL001689	Robinia pseudoacacia	5	Platycodi radix
MOL007050	2-(4-hydroxy-3-methoxyphenyl)-5-(3-hydroxypropyl)-7-methoxy-3-benzofuran formaldehyde	2	*Salvia miltiorrhiza*

**Table 4:** Component-target molecular docking.

Target protein	Binding energy/(kJ·Mol^−1^)					
	Quercetin	Luteolin	Baicalein	Kaempferol	Tanshinone IIA	Cryptotanshinone
AKT1	-8.8	-8.3	-8	-8.6		
NFKBIA	-8.4	-9			-8.8	
CCL2	-7.6					
IL1B	-6.7					
STAT3						-9
ICAM1	-6.4	-6.9		-6.6		
EGF	-6.9					
TP53	-6.3	-6.4	-6.3		-7	
EGFR	-8	-7.9				
FOS		-9.2	-7.9		-8.4	
CASP3	-7.9	-7.8	-7.4	-7.7	-8.7	
PTGS2	-9.1	-9.2	-8.9	-8.8	-9.3	-9.7
JUN	-8.2	-8.1		-9.4	-8.5	
MMP9	-9.7	-9.9	-9.7		-8.4	
IL6	-7	-7.1				
TNF	-5.2	-5.1		-5.1		-5.9
HIF1A	-7.4		-7			
VEGFA	-7	-7	-6.8			
MYC	-5.6				-6.2	

## Data Availability

The datasets used and/or analysed during the current study available from the corresponding author on reasonable request.

## Supplementary Materials

Supplementary Materials Supplementary Table 1. Active components of Shufeiya recipe.
