# Supplementary material for: Network Pharmacology and Molecular Docking Analysis of Shufeiya Recipe in the Treatment of Pulmonary Hypertension
Source: Biomed Res Int. 2022 Dec 28;2022:7864976. doi: 10.1155/2022/7864976 (PMC9900250; doi:10.1155/2022/7864976)
Supplement: Supplementary Materials — Supplementary Table 1. Active components of Shufeiya recipe. [file 7864976.f1.docx]

**Supplementary Table 1.** Active components of Shufeiya recipe

| Drug | MOL ID | NAME | MW | Allogp | Hdon | Hacc | OB | DL |
| --- | --- | --- | --- | --- | --- | --- | --- | --- |
| Salvia miltiorrhiza | MOL001601 | 1,2,5,6-tetrahydrotanshinone | 280.34 | 2.98 | 0 | 3 | 38.8 | 0.36 |
| Salvia miltiorrhiza | MOL001942 | isoimperatorin | 270.3 | 3.65 | 0 | 4 | 45.5 | 0.23 |
| Salvia miltiorrhiza | MOL002222 | sugiol | 300.48 | 4.99 | 1 | 2 | 36.1 | 0.28 |
| Salvia miltiorrhiza | MOL002651 | Dehydrotanshinone II A | 292.35 | 4.22 | 0 | 3 | 43.8 | 0.4 |
| Salvia miltiorrhiza/safflower/platycodi radix | MOL000006 | luteolin | 286.25 | 2.07 | 4 | 6 | 36.2 | 0.25 |
| Salvia miltiorrhiza | MOL007036 | 5,6-dihydroxy-7-isopropyl-1,1-dimethyl-2,3-dihydrophenanthren-4-one | 298.41 | 4.38 | 2 | 3 | 33.8 | 0.29 |
| Salvia miltiorrhiza | MOL007041 | 2-isopropyl-8-methylphenanthrene-3,4-dione | 264.34 | 4.16 | 0 | 2 | 40.9 | 0.23 |
| Salvia miltiorrhiza | MOL007045 | 3α-hydroxytanshinoneⅡa | 310.37 | 3.56 | 1 | 4 | 44.9 | 0.44 |
| Salvia miltiorrhiza | MOL007048 | (E)-3-[2-(3,4-dihydroxyphenyl)-7-hydroxy-benzofuran-4-yl]acrylic acid | 312.29 | 3.21 | 4 | 6 | 48.2 | 0.31 |
| Salvia miltiorrhiza | MOL007049 | 4-methylenemiltirone | 266.36 | 4.33 | 0 | 2 | 34.4 | 0.23 |
| Salvia miltiorrhiza | MOL007050 | 2-(4-hydroxy-3-methoxyphenyl)-5-(3-hydroxypropyl)-7-methoxy-3-benzofurancarboxaldehyde | 356.4 | 3.58 | 2 | 6 | 62.8 | 0.4 |
| Salvia miltiorrhiza | MOL007058 | formyltanshinone | 290.28 | 3.36 | 0 | 4 | 73.4 | 0.42 |
| Salvia miltiorrhiza | MOL007059 | 3-beta-Hydroxymethyllenetanshiquinone | 294.32 | 3.16 | 1 | 4 | 32.2 | 0.41 |
| Salvia miltiorrhiza | MOL007061 | Methylenetanshinquinone | 278.32 | 4.26 | 0 | 3 | 37.1 | 0.36 |
| Salvia miltiorrhiza | MOL007063 | przewalskin a | 398.49 | 2.25 | 1 | 6 | 37.1 | 0.65 |
| Salvia miltiorrhiza | MOL007064 | przewalskin b | 330.46 | 3.18 | 1 | 4 | 110 | 0.44 |
| Salvia miltiorrhiza | MOL007068 | Przewaquinone B | 292.3 | 2.99 | 1 | 4 | 62.2 | 0.41 |
| Salvia miltiorrhiza | MOL007069 | przewaquinone c | 296.34 | 3.31 | 1 | 4 | 55.7 | 0.4 |
| Salvia miltiorrhiza | MOL007070 | (6S,7R)-6,7-dihydroxy-1,6-dimethyl-8,9-dihydro-7H-naphtho[8,7-g]benzofuran-10,11-dione | 312.34 | 2.34 | 2 | 5 | 41.3 | 0.45 |
| Salvia miltiorrhiza | MOL007071 | przewaquinone f | 312.34 | 2.07 | 2 | 5 | 40.3 | 0.46 |
| Salvia miltiorrhiza | MOL007077 | sclareol | 308.56 | 4.27 | 2 | 2 | 43.7 | 0.21 |
| Salvia miltiorrhiza | MOL007079 | tanshinaldehyde | 308.35 | 3.83 | 0 | 4 | 52.5 | 0.45 |
| Salvia miltiorrhiza | MOL007081 | Danshenol B | 354.48 | 2.59 | 1 | 4 | 58 | 0.56 |
| Salvia miltiorrhiza | MOL007082 | Danshenol A | 336.41 | 2.01 | 1 | 4 | 57 | 0.52 |
| Salvia miltiorrhiza | MOL007085 | Salvilenone | 292.4 | 4.26 | 0 | 2 | 30.4 | 0.38 |
| Salvia miltiorrhiza | MOL007088 | cryptotanshinone | 296.39 | 3.44 | 0 | 3 | 52.3 | 0.4 |
| Salvia miltiorrhiza | MOL007093 | dan-shexinkum d | 336.41 | 2.83 | 1 | 4 | 38.9 | 0.55 |
| Salvia miltiorrhiza | MOL007094 | danshenspiroketallactone | 282.36 | 3.24 | 0 | 3 | 50.4 | 0.31 |
| Salvia miltiorrhiza | MOL007098 | deoxyneocryptotanshinone | 298.41 | 4.32 | 1 | 3 | 49.4 | 0.29 |
| Salvia miltiorrhiza | MOL007100 | dihydrotanshinlactone | 266.31 | 2.77 | 0 | 3 | 38.7 | 0.32 |
| Salvia miltiorrhiza | MOL007101 | dihydrotanshinoneⅠ | 278.32 | 2.86 | 0 | 3 | 45 | 0.36 |
| Salvia miltiorrhiza | MOL007105 | epidanshenspiroketallactone | 284.38 | 2.37 | 0 | 3 | 68.3 | 0.31 |
| Salvia miltiorrhiza | MOL007108 | isocryptotanshi-none | 296.39 | 3.59 | 0 | 3 | 55 | 0.39 |
| Salvia miltiorrhiza | MOL007111 | Isotanshinone II | 294.37 | 4.66 | 0 | 3 | 49.9 | 0.4 |
| Salvia miltiorrhiza | MOL007119 | miltionone Ⅰ | 312.39 | 3.33 | 1 | 4 | 49.7 | 0.32 |
| Salvia miltiorrhiza | MOL007120 | miltionone Ⅱ | 312.39 | 2.14 | 1 | 4 | 71 | 0.44 |
| Salvia miltiorrhiza | MOL007121 | miltipolone | 300.43 | 2.74 | 1 | 3 | 36.6 | 0.37 |
| Salvia miltiorrhiza | MOL007122 | Miltirone | 282.41 | 4.73 | 0 | 2 | 38.8 | 0.25 |
| Salvia miltiorrhiza | MOL007124 | neocryptotanshinone ii | 270.35 | 3.61 | 1 | 3 | 39.5 | 0.23 |
| Salvia miltiorrhiza | MOL007125 | neocryptotanshinone | 314.41 | 3.01 | 2 | 4 | 52.5 | 0.32 |
| Salvia miltiorrhiza | MOL007127 | 1-methyl-8,9-dihydro-7H-naphtho[5,6-g]benzofuran-6,10,11-trione | 280.29 | 3.21 | 0 | 4 | 34.7 | 0.37 |
| Salvia miltiorrhiza | MOL007130 | prolithospermic acid | 314.31 | 2.77 | 4 | 6 | 64.4 | 0.31 |
| Salvia miltiorrhiza | MOL007132 | (2R)-3-(3,4-dihydroxyphenyl)-2-[(Z)-3-(3,4-dihydroxyphenyl)acryloyl]oxy-propionic acid | 360.34 | 2.69 | 5 | 8 | 109 | 0.35 |
| Salvia miltiorrhiza | MOL007141 | salvianolic acid g | 340.3 | 2.2 | 4 | 7 | 45.6 | 0.61 |
| Salvia miltiorrhiza | MOL007143 | salvilenone Ⅰ | 270.4 | 2.88 | 1 | 2 | 32.4 | 0.23 |
| Salvia miltiorrhiza | MOL007145 | salviolone | 268.38 | 4.05 | 1 | 2 | 31.7 | 0.24 |
| Salvia miltiorrhiza | MOL007150 | (6S)-6-hydroxy-1-methyl-6-methylol-8,9-dihydro-7H-naphtho[8,7-g]benzofuran-10,11-quinone | 312.34 | 2.42 | 2 | 5 | 75.4 | 0.46 |
| Salvia miltiorrhiza | MOL007151 | Tanshindiol B | 312.34 | 2.34 | 2 | 5 | 42.7 | 0.45 |
| Salvia miltiorrhiza | MOL007152 | Przewaquinone E | 312.34 | 2.34 | 2 | 5 | 42.9 | 0.45 |
| Salvia miltiorrhiza | MOL007154 | tanshinone iia | 294.37 | 4.66 | 0 | 3 | 49.9 | 0.4 |
| Salvia miltiorrhiza | MOL007155 | (6S)-6-(hydroxymethyl)-1,6-dimethyl-8,9-dihydro-7H-naphtho[8,7-g]benzofuran-10,11-dione | 310.37 | 3.57 | 1 | 4 | 65.3 | 0.45 |
| Salvia miltiorrhiza | MOL007156 | tanshinone Ⅵ | 296.34 | 2.44 | 2 | 4 | 45.6 | 0.3 |
| Safflower | MOL002694 | 4-[(E)-4-(3,5-dimethoxy-4-oxo-1-cyclohexa-2,5-dienylidene)but-2-enylidene]-2,6-dimethoxycyclohexa-2,5-dien-1-one | 356.4 | 0.36 | 0 | 6 | 48.5 | 0.36 |
| Safflower | MOL002695 | lignan | 458.55 | 3.78 | 0 | 8 | 43.3 | 0.65 |
| Safflower | MOL002710 | Pyrethrin II | 372.5 | 3.74 | 0 | 5 | 48.4 | 0.35 |
| Safflower | MOL002712 | 6-Hydroxykaempferol | 302.25 | 1.5 | 5 | 7 | 62.1 | 0.27 |
| Safflower | MOL002714 | baicalein | 270.25 | 2.33 | 3 | 5 | 33.5 | 0.21 |
| Safflower | MOL002717 | qt_carthamone | 286.25 | 0.7 | 3 | 6 | 51 | 0.2 |
| Safflower | MOL002757 | 7,8-dimethyl-1H-pyrimido[5,6-g]quinoxaline-2,4-dione | 242.26 | 0.59 | 2 | 6 | 45.8 | 0.19 |
| Safflower | MOL000422 | kaempferol | 286.25 | 1.77 | 4 | 6 | 41.9 | 0.24 |
| Safflower | MOL000098 | quercetin | 302.25 | 1.5 | 5 | 7 | 46.4 | 0.28 |
| Platycodi radix | MOL001689 | acacetin | 284.28 | 2.59 | 2 | 5 | 35 | 0.24 |
| Platycodi radix | MOL004580 | cis-Dihydroquercetin | 304.27 | 1.49 | 5 | 7 | 66.4 | 0.27 |
| Cornus officinalis | MOL005503 | Cornudentanone | 378.56 | 4.97 | 0 | 5 | 39.7 | 0.33 |
| Cornus officinalis | MOL005530 | Hydroxygenkwanin | 300.28 | 2.32 | 3 | 6 | 36.5 | 0.27 |
| Cornus officinalis | MOL005531 | Telocinobufagin | 402.58 | 2.11 | 3 | 5 | 70 | 0.79 |
| Cornus officinalis | MOL008457 | Tetrahydroalstonine | 352.47 | 2.66 | 1 | 4 | 32.4 | 0.81 |
